# Conductive Polymer (Graphene/PPy)–BiPO_4_ Composite Applications in Humidity Sensors

**DOI:** 10.3390/polym13122013

**Published:** 2021-06-20

**Authors:** Zhen Zhu, Wang-De Lin, Zhi-Yi Lin, Ming-Hong Chuang, Ren-Jang Wu, Murthy Chavali

**Affiliations:** 1 School of Environmental Science and Safety Engineering, Tianjin University of Technology, Tianjin 300384, China; zhenzhu@tjut.edu.cn; 2 Department of Center for General Education, St. Mary’s Junior College of Medicine Nursing and Management, Yilan 26647, Taiwan; 3 Department of Applied Chemistry, Providence University, Taichung 43301, Taiwan; s1063053@gm.pu.edu.tw (Z.-Y.L.); g1096043@gm.pu.edu.tw (M.-H.C.); rjwu@pu.edu.tw (R.-J.W.); 4 NTRC-MCETRC and Aarshanano Composite Technologies Pvt. Ltd., Guntur District, Andhra Pradesh 522201, India; Chavalim@gmail.com

**Keywords:** graphene, polypyrrole, impedance, BiPO4, sensing response

## Abstract

In this particular experiment, a chain of conductive polymer graphene/polypyrrole (Gr/PPy) and BiPO_4_—or (Gr/PPy)–BiPO_4_—materials were prepared and used as moisture-sensitive materials. The structure and morphology of the conductive polymer (Gr/PPy)–BiPO_4_ materials were analyzed using an X-ray diffractometer, scanning electron microscopy, transmission electron microscopy, and energy-dispersive X-ray spectroscopy. Moreover, properties such as hysteresis loop, impedance, sensing response, and response and recovery time were calculated and evaluated using an inductance–capacitance–resistance analyzer. The data expressed that PPy/BiPO_4_, as prepared in this study, exhibited excellent sensing properties, with impedance changing by only a few orders of range. Furthermore, the response time and time of recovery were 340 s and 60 s, respectively, and negligible humidity hysteresis occurred at different relative humidities. Therefore, conductive PPy/BiPO_4_, as prepared in the present study, is an excellent candidate for application in humidity sensors.

## 1. Introduction

Ambient humidity must be controlled, regulated, and monitored in environments such as dry cabinets, warehouse storage, industrial production, and food processing facilities, as well as in agricultural planting operations, medical diagnostic centers—both facilities in which high-tech instruments are employed—and shelter environments designed for managing life [1,2]. Recently, tremendous scientific effort has been devoted to the detection of environmental humidity. Particularly, many types of humidity sensors have been explored due to their advantages, such as high sensitivity, rapid response and recovery, low hysteresis, and excellent reproducibility. Humidity sensors are commonly classified into capacitive [3,4], resistive [5,6], optical [7,8], bulk acoustic wave [9,10], and quartz crystal microbalance [11,12] humidity sensors. Numerous types of humidity-sensing material have been used for humidity detection. Zhao et al. prepared a SnO_2_/MoS_2_ hybrid-sensing nanocomposite by synthesis through a two-step hydrothermal route; the sensor with a 5 μm gap had the largest sensitivity at low humidity [13]. Mallick et al. enhanced the humidity-sensing properties of polyvinylidene fluoride titanium dioxide (PVDF-TiO_2_) nanocomposites-based capacitive humidity sensors by modifying the film surface of the TiO_2_ [14]. Duong et al. fabricated composite multi-walled carbon nanotubes (MWCNTs) and tungsten oxide (WO_3_) nanobricks at different mass ratios, which attained a high response with 95 wt.% of MWCNT/WO_3_ nanocomposite [15]. Arularasu et al. developed a PVDF/ZnO nanocomposite that was prepared as a humidity sensor through a simple hydrothermal approach in which the PVDF/ZnO provided more water molecule adsorption and desorption across the humidity sensor surface compared to pure ZnO nanoparticles [16]. Using the cast-drop and evaporation technique, Zebian et al. developed a hydrophilic-sensitive film based on Al_2_O_3_-PVA that was coated on the surface of no-core fibers; this exhibited high sensitivity to Al_2_O_3_ [17]. Ye et al. used Fe_3_O_4_ nanoparticles as an adsorption interface for the concurrent removal of gaseous benzene, toluene, ethylbenzene, m-xylene, and sulfur dioxide at different relative humidities, which indicated high removal efficiencies under dry conditions of Fe_3_O_4_ [18]. Xie et al. used the complex impedance analysis method to analyze CeO_2_ nanoparticles (NPs), which exhibited a rapid and reversible response characterized by a very small hysteresis [19]. Manikandan et al. synthesized nanocrystalline lithium-substituted copper ferrite (Lisingle bond CuFe_2_O_4_) nanoparticles with a high surface area, and obtained a fast response/recovery time [20]. Manikandan et al. synthesized a nanocrystalline-structured tin-substituted nickel ferrite (Sn-NiFe_2_O_4_) thin film, which exhibited excellent reproducibility, sensitivity, and response and recovery times [21]. Douani et al. synthesized bismuth ferrite nanoparticles BiFeO_3_ and bismuth ferrite/carbon fiber nanocomposites (BFO/CFs) by a hydrothermal process, showing that they have small hysteresis and good stability [22]. Arrizabalaga et al. reported on an accurate interferometric fiber optic humidity sensor of miniature size which showed fast, accurate, and sensitive properties as a commercial capacitive humidity sensor [23]. Hu et al. fabricated a novel, highly stable and sensitive humidity sensor based on bacterial cellulose (BC)-coated quartz crystal microbalance, which exhibited good reversible behavior and good long-term stability [24]. Ko et al. first report all-two-dimensional (2D) bis (trifluoromethane sulfonyl)-amide (TFSA)-doped graphene (GR) (TFSA-GR)/MoS_2_/ triethylenetetramine (TETA)-doped GR (TETA-GR) vertical-heterostructure semitransparent photodetectors (PDs) on rigid/flexible substrates, which exhibited good stability [25]. Among these, polymers are promising humidity-sensing candidates because they exhibit dramatic changes in electrical conductivity when exposed to various levels of environmental humidity [26,27,28]; they also have excellent properties, are low-cost to prepare, nontoxic, easy to fabricate, and are stable at room temperature. Additionally, graphene-based humidity sensors have received increased attention because they can generate electricity from the air by absorbing water molecules [29,30,31].

BiPO_4_ is an attractive material to researchers due to its economic cost, chemically stable structure, nontoxicity, photocatalytic characteristics, extraordinary optics, and electronic properties [32,33]. Graphene has attracted much attention from scientists and researchers because of its outstanding advantages, such as high surface area, excellent thermal conductivity, low cost, large-scale synthesis, stability, and electrical and mechanical properties; however, graphene cannot be applied to humidity sensors because of its hydrophobic nature. However, no attempt has been made to construct graphene/polypyrrole (Gr/PPy)–BiPO_4_ composite-resistive humidity sensors. In the present research, a series of Gr/PPy-modified BiPO_4_ was successfully synthesized, characterized, and investigated as sensing materials for humidity sensors. Moreover, the sensing mechanism, sensing properties, and morphology of as-prepared Gr/PPy-modified BiPO_4_ were examined.

## 2. Experimental Design

### 2.1. Materials

The 97% pure (Bi(NO_3_)_3_·5H_2_O) bismuth(III)nitrate pentahydrate, trisodium phosphate (Na_3_PO_4_), pyrrole (C_4_H_5_N), cetyltrimethylammonium bromide (CTAB), ammonium persulfate (NH_4_)_2_S_2_O_8_), and polyvinyl alcohol ((C_2_H_4_O)_x_) were obtained from Sigma-Aldrich Co., Inc. (St. Louis, MO, USA). Graphene was purchased from National Chiayi University in Taiwan. All materials were utilized without any purification. DI water was drawn from a system water purification provided by the Milli-Q system, processed, and used in all experiments.

### 2.2. Method of Synthesis

As is typical in this procedure, 5 mmol of Bi(NO_3_)_3_·5H_2_O and 2.5 mmol of Na_3_PO_4_ were dissolved in 46 mL of HNO_3_ (0.3 M) under continuous stirring for 1 h to obtain a homogeneous aqueous solution. The resulting solution was transferred into a Teflon-lined stainless steel autoclave. Subsequently, the autoclave was tightly sealed and maintained in a temperature-controlled electric oven at 160 °C for 24 h. Then, after it was filtered and washed with deionized water and ethanol five times, the resulting white product was dried at 70 °C for 12 h to harvest BiPO_4_. Next, an amount of 0.5 g as-prepared BiPO_4_ was added to 300 mL of an aqueous solution containing 1% CTAB under ultrasound for 0.5 h to form solution A, whereas solution B was formed by dissolving 0.03 mL of C_4_H_5_N in solution A under ultrasound for 10 min. Thereafter, 100 mL of 10% (NH_4_)_2_S_2_O_8_ and solution B were mixed under ultrasound for 0.5 h and maintained under temperatures ranging from 1 °C to 5 °C for 24 h to form a black precipitate. Finally, the as-obtained precipitate was centrifuged, washed with deionized water and ethanol several times, and dried at 70 °C for 2 h to obtain PPy/BiPO_4_. Additionally, precalculated amounts of as-prepared BiPO4 and graphene were added to 10 mL of 95% ethanol under ultrasound for 1 h to produce a black suspension. The obtained suspension was centrifuged and washed with ethanol and deionized water and then dried at 60 °C for 12 h to obtain graphene/BiPO_4_ [34,35,36].

### 2.3. Characteristic Methods

Structural properties of as-prepared material were analyzed using the powder X-ray diffractometer (XRD) method—the Shimadzu XRD-6000 (operated at 35 kV and 35 mA) with a (λ = 1.5404 Å) Cu source. The particle size of the as-obtained prepared photocatalysts was averaged and explained using the Debye–Scherrer equation. Transmission electron microscopy (TEM) images were acquired using a JEOL JEM2010 transmission electron microscope (JEOL, Japan) at an accelerating voltage of 200 kV. The samples for TEM analysis were suspended in ethanol with the assistance of ultrasound and dispersed on a copper grid. The chemical composition of the as-synthesized samples was analyzed using an energy-dispersive X-ray spectroscope (EDS). The morphology of different as-prepared materials was observed using field emission scanning electron microscopy (JEOL JSM-7100F, Tokyo, Japan), with the microscope operated at 30 kV.

### 2.4. Sensor Fabrication and Humidity Testing

A solution of 10% PVA was used as the binder. The sensing chips were fabricated by dip-coating them on a pair of comb-like gold electrodes on an alumina substrate (10 × 5 mm^2^; rotational speed, 1000 rpm). The chips were subsequently heated at 80 °C for 0.5 h and calcined at 300 °C for 4 h.

**Figure 1:**
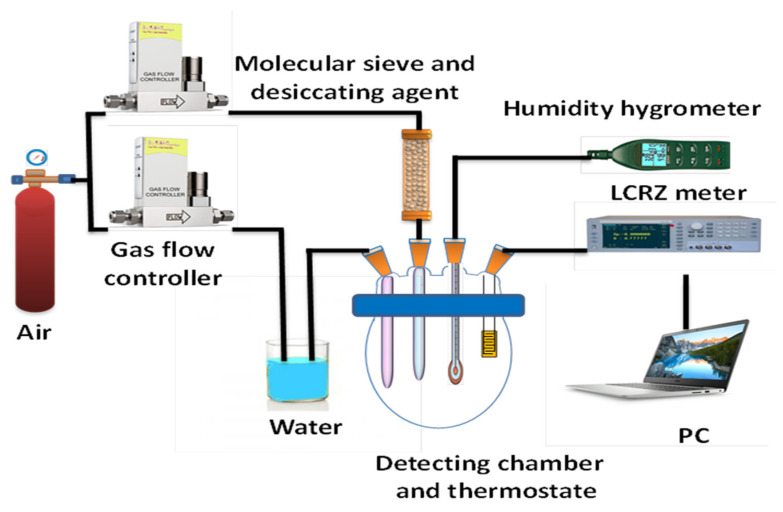
Schematic diagram of the experimental setup.

The humidity parameter and its response were calculated in a flow system that worked dynamically [37], wherein an airtight glass chamber was developed to preserve the sensors and store them (Figure 1). In the sensor setup, air was injected into the water to generate water vapor, which subsequently filled the testing chamber. Various levels of specific relative humidity (RH) were maintained for 15 min to enable the humidity sources to reach equilibrium. The chamber temperature was controlled at 25 °C ± 2 °C; a thermos-hygrometer was connected to the testing chamber for RH measurement. Taiwan’s Center of Measurement Standard/ITRI provides the standard concentration which was used to calibrate the commercial sensor for humidity. The RH response (S) sensor was calculated as S equals the ratio of Rd and Rh. The parameters of Rd under dry conditions were measured as a value of resistance (12% RH) for the developed sensor, while Rh was measured according to how much resistance would be exerted at a specific humidity [36]. The humidity hysteresis properties were 12% and 90%, and they were then decreased to 12% for the adsorption and desorption of water molecules [38]. H = Δfmax/ffs × 100% was evaluated as humidity hysteresis error (H) using the equation provided above, wherein Δfmax was the error of hysteresis at its maximum and ffs was the output for the response of the full scale. The response or recovery time was defined as the time required for the impedance of a sensor to change by 90% of the total impedance, whereas the recovery time was the time required for the reverse process. For dynamic testing, the changes in RH at 12% and 90% were controlled by feeding different ratios of air to water. In the provided experiment setup, a hygrometer (Rotronic) was utilized for measuring the RH value with ±0.1% RH accuracy. Sensing material for the response of impedance against humidity was explained by an analyzer that could measure chemical impedance (DU 6010); the input voltage and frequency were 1 V and 1 kHz, respectively.

## 3. Results and Discussion

### 3.1. Material Characterizations

**Figure 2:**
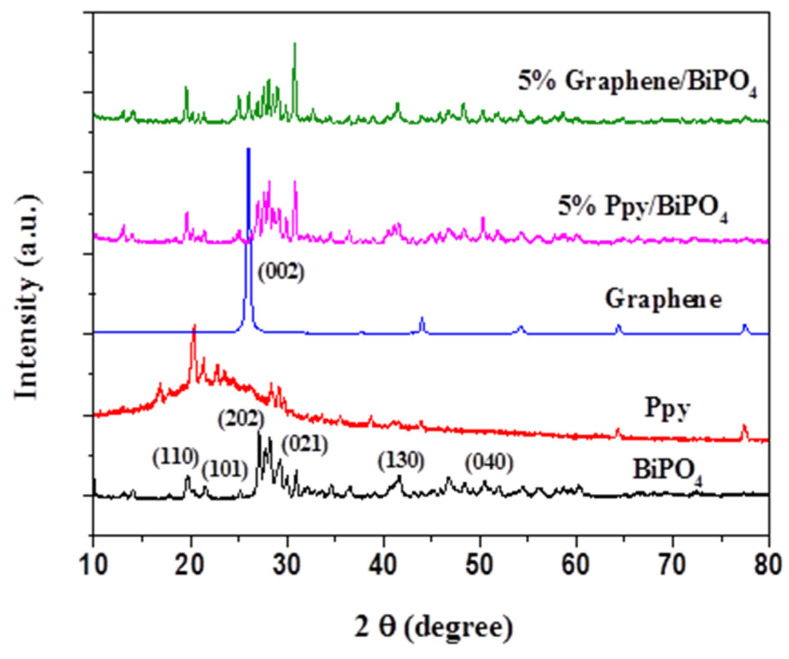
XRD pattern of BiPO_4_, PPy, graphene, 5 wt.% PPy/BiPO_4_, and 5 wt.% graphene/BiPO_4_ composites.

To investigate the structural features of the as-fabricated samples, XRD measurement was performed. The XRD patterns of the samples presented in Figure 2 indicate that the main diffraction peak positions were located at the (110), (101), (202), (021), (130), and (040) crystal planes of BiPO_4_, at 2θ values of 21.3°, 23.4°, 27.6°, 41.5° and 50.9° [39], respectively. Additionally, the diffraction peaks were observable at 2θ values of 22.3° and 26.8°, which corresponded to the crystalline planes of PPy and graphene [40,41]. These results indicated that PPy/BiPO_4_ and Gr/BiPO_4_ were successfully synthesized. The diffraction peaks of BiPO_4_ with the main lattice plane are (101), (200), (102), and (211) compared to JCPDS, which demonstrate a similar hexagonal phase.

**Figure 3:**
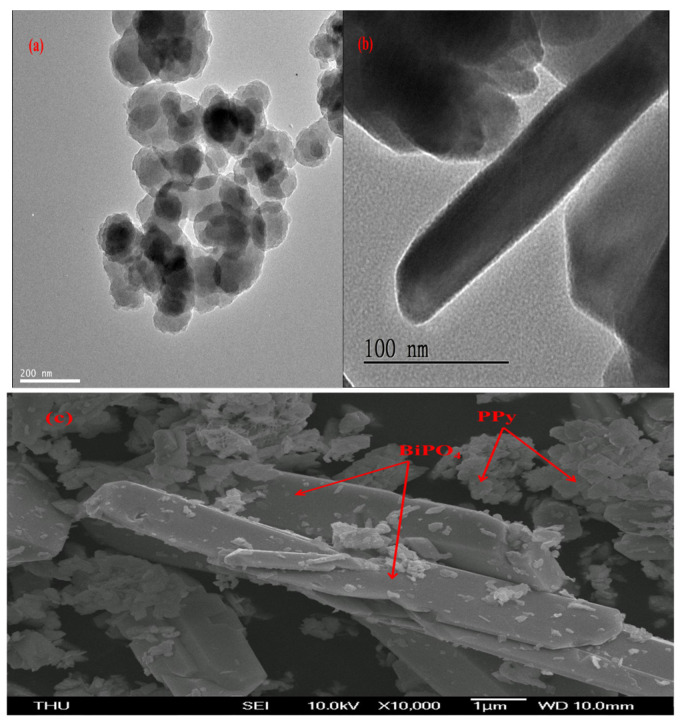
TEM images of (**a**) PPy, (**b**) BiPO_4_, and (**c**) FESEM 5 wt.% PPy/BiPO_4_ composites.

**Figure 4:**
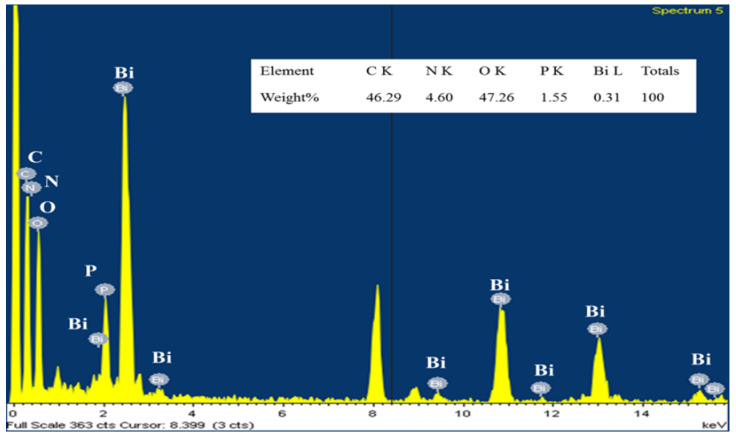
EDX spectrum of the 5 wt.% PPy/BiPO_4_ composites.

Surface morphology plays an essential role in the humidity-sensing properties of sensing materials, as illustrated in Figure 3a–c. Figure 3a presents polymer diameters roughly in the range of 100 to 150 nanometers for domains in PPy. Figure 3b shows the TEM image of the BiPO_4_ (200) lattice plane, which is about 0.329 nm in terms of lattice displacement. TEM images of BiPO_4_ are shown at low magnification, at a scale of 100 nm, as well as at a higher magnification. The figure also shows a TEM image of a single BiPO_4_ nanorod 569.53 nm in length and 97.92 nm in width. Figure 3c illustrates that leaf-like nanoparticles were uniformly and thinly coated on the surface of the rod-like nanoparticles. Figure 4 suggests that the elemental analysis (EDS) spectrum for PPy/BiPO_4_ indicated the presence of C, O, N, P, and Bi atoms as major components in the as-prepared PPy/BiPO_4_.

### 3.2. Sensing Humidity

**Figure 5:**
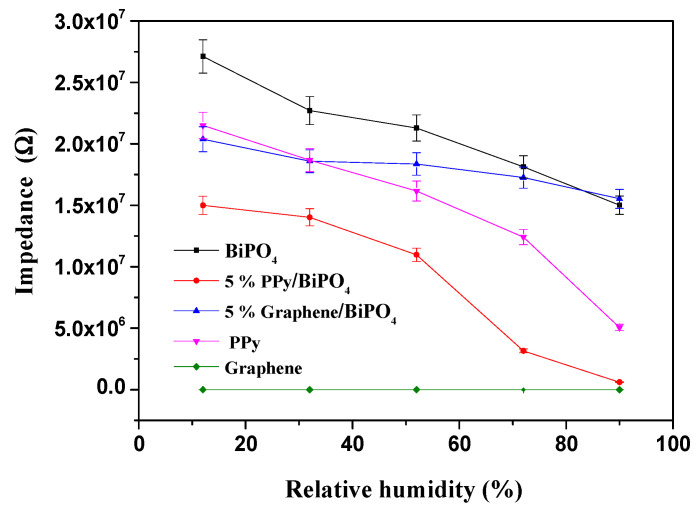
Discrepancies in impedance relative to the variations in relative humidity (%) for varying (Gr/PPy)-BiPO_4_ composite contents.

The roles of the as-prepared samples in the humidity control were measured and investigated at the optimal operating frequency (1 kHz) (Figure 5). Figure 5 indicates that the impedance values of the as-prepared samples (BiPO_4_, PPy, Gr, PPy/BiPO_4_, and Gr/BiPO_4_) were inversely related to RH changes. Additionally, PPy/BiPO_4_ exhibited the most significant change within the RH range, from 12% to 90% (a change of almost three orders of magnitude), suggesting that PPy/BiPO_4_ can absorb more water molecules and is thus more sensitive to humidity.

**Figure 6:**
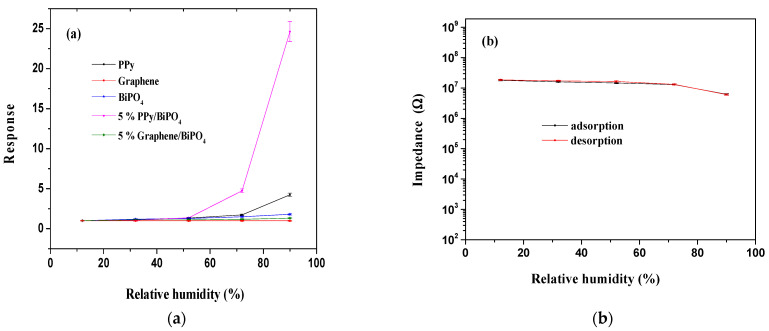
(**a**) Discrepancies in response relative to the variations in relative humidity (%) for varying (Gr/PPy)-BiPO_4_ composite contents; (**b**) hysteresis characteristics of 5 wt.% PPy/BiPO_4_ composite.

Figure 6a suggests that the response of the as-prepared sample changed gradually with increasing RH under humidity levels lower than 50% RH, whereas the response of the sensor increased sharply with higher ranges of RH. Moreover, PPy/BiPO_4_ exhibited the highest humidity in response (S = 24.6) to changes in RH (Figure 6).

Humidity hysteresis, defined as the maximum difference in measured values of RH when the humidity sensor is exposed to adsorption and desorption processes, is an essential parameter for evaluating the reliability of a humidity sensor. Figure 6b suggests that PPy/BiPO_4_ had minor humidity hysteresis loss and a relatively large hysteresis loop (0.70%) that occurred at RH levels less than 50%; this indicated that a fast equilibrium could be achieved between the adsorption and desorption process for PPy/BiPO_4_.

**Figure 7:**
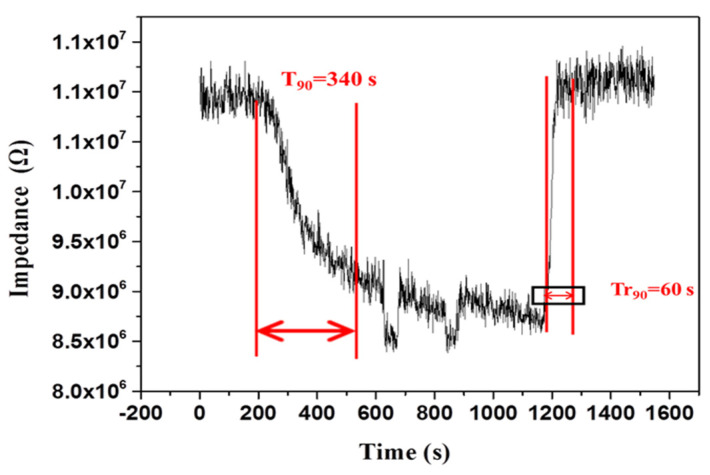
Response and recovery characteristics of 5 wt.% PPy/BiPO_4_ composites.

**Figure 8:**
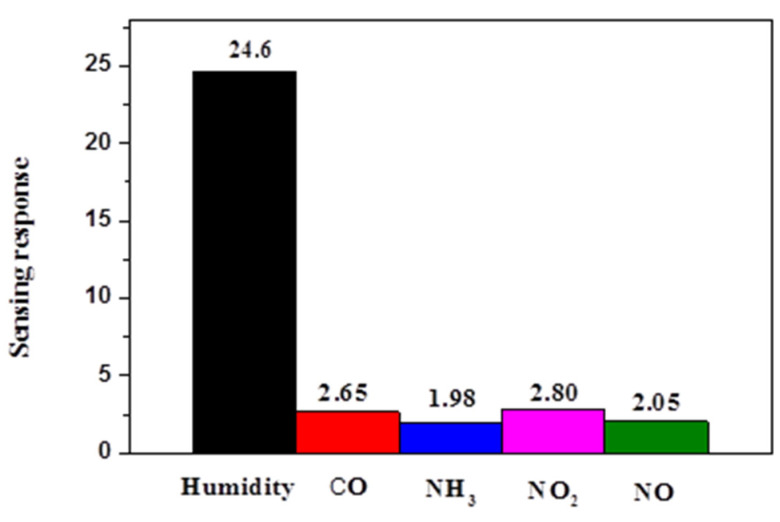
Selectivity towards relative humidity and various gas species of 5 wt.% PPy/BiPO_4_ composites.

Crucial parameters such as time of recovery and response data are vital for evaluating the performance of humidity sensors. These parameters represent humidity sensors’ speed in measuring RH in various environments. Figure 7 illustrates that the response and recovery times for PPy/BiPO_4_ were 340 s and 60 s between 12% RH and 90% RH, respectively. The observed sensing parameters were compared with the previously reported sensors [4,42,43,44,45] listed in Table 1. Overall, the PPy/BiPO_4_ sensor exhibited an excellent sensing response across the whole humidity range; however, its response and recovery times were longer than those of other sensors, as the PPy/BiPO_4_ sensor was tested using materials with different sensitivities. Selectivity is an essential parameter for the practical application of humidity sensors. The cross-sensitivity of PPy/BiPO_4_ has been studied for various gases at room temperatures, such as carbon monoxide (CO), ammonia (NH_3_), nitrogen dioxide (NO_2_), and nitric oxide (NO). As Figure 8 illustrates, the PPy/BiPO_4_ sensor had the highest selectivity for humidity and the lowest responsiveness to other types of gases.

### 3.3. Humidity-Sensing Mechanism

**Figure 9:**
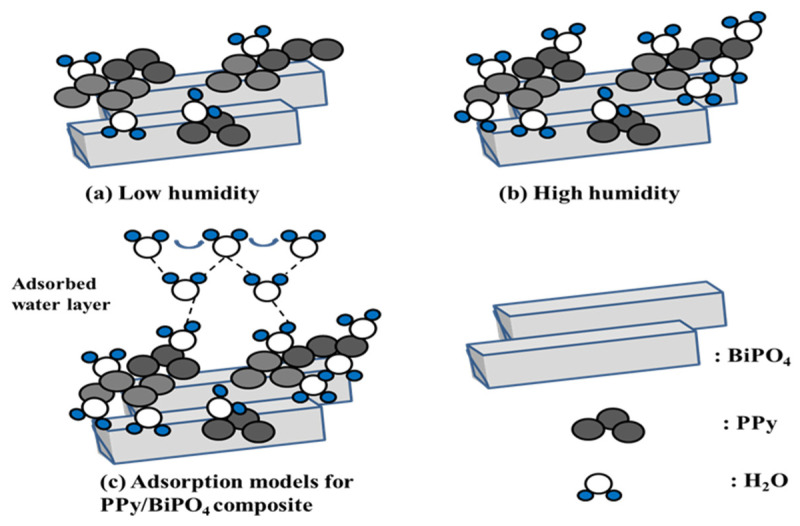
Scheme of humidity-sensing mechanism: (**a**) low humidity, (**b**) high humidity, (**c**) adsorption models for PPy/BiPO_4_ composites.

The humidity-sensing mechanism can be explained by the chemical and physical adsorption of water molecules on the PPy/BiPO_4_ surface, as illustrated in Figure 9. At low humidity, the probability of contact between water molecules and PPy/BiPO_4_ composite was low, so only the outer particles came into contact with the water molecules, as shown in Figure 9a. Since water molecules could not form a continuous water layer in this process, the transfer of H_2_O or H_3_O^+^ onto the discontinuous water layer was challenging [46]. Therefore, in addition to its use of high-conductor graphene, the sensor impedance is extremely high. Figure 5 shows that, when the RH was 12% in the pure PPy, pure graphene and pure BiPO_4_, Gr/BiPO_4_, and PPy/BiPO_4_ composite, the impedance was 2.15 × 10^7^ Ω, 9.18 × 10^–^^1^ Ω, 2.71 × 10^7^ Ω, 2.03 × 10^7^ Ω, 1.49 × 10^7^ Ω, respectively. As the RH level increased, the physisorption of water molecules occurred on the chemisorbed layer through hydrogen bonding, and the formation of a water multilayer, as illustrated in Figure 9b. The serial water layers accelerated the transfer of H_2_O or H_3_O^+^. The ion transfer mechanism presented by Grotthuss [47] and Casalbore-Miceli et al. [48] involves the transfer of H_2_O or H_3_O^+^ on serial water layers H_2_O + H_3_O^+^ → H_3_O^+^ + H_2_O, as shown in Figure 9c. An ionic transfer is the main conduction mode in this process, and the impedance decreases as the relative humidity increases. The rapid transfer of ions on the water layer sharply reduces impedance. The PPy/BiPO_4_ composites exhibited humidity-sensing properties that are more favorable than those of the pure PPy or BiPO_4_ samples.

## 4. Conclusions

In this paper, various polymer (Gr/PPy)–BiPO_4_ materials were successfully synthesized and used to detect RH. The structure and morphology samples were characterized by XRD, TEM, FESEM, and EDX. In this study, a humidity sensor based on Gr/BiPO_4_ and PPy/BiPO_4_ was prepared under room temperature conditions. The experimental results show that the PPy/BiPO_4_ composite exhibited excellent humidity-sensing capabilities, including negligible humidity hysteresis, sensing response (S = 24.6), and high selectivity. Moreover, the response was 340 s and the recovery time was 30 s. Compared to pure PPy, pure graphene, and pure BiPO_4_, as well as Gr/BiPO_4_ and PPy/BiPO_4_ composites, these results indicate that PPy/BiPO_4_-based humidity sensors are good candidates for humidity sensor applications.

## Figures and Tables

**Table 1:** Comparative humidity-sensing performance of modified conductive polymer-based sensors with previously published reports.

Sensing Material	Measurement Range (% RH)	Response/Recovery Time (s)	References
MCM-41/PPy	11–95	−915/−100	[42]
RGO/SnO_2_	11–97	102/6	[43]
Trianglamine hydrochloride	5–95	720/300	[44]
MCM-41/PEDOT	11–95	165/115	[4]
PPy	11–95	41/120	[45]
PPy/BiPO_4_	12–90	340/60	This work

## Data Availability

Data sharing not applicable.
